# Triage capabilities of medical trainees in Ghana using the South African triage scale: an opportunity to improve emergency care

**DOI:** 10.11604/pamj.2016.24.294.8728

**Published:** 2016-08-03

**Authors:** Adam Gyedu, Kwabena Agbedinu, Mohammed Dalwai, Maxwell Osei-Ampofo, Emmanuel Kweku Nakua, Rockefeller Oteng, Barclay Stewart

**Affiliations:** 1 Department of Surgery, School of Medical Sciences, Kwame Nkrumah University of Science and Technology, Kumasi, Ghana; 2 Directorate of Surgery, Komfo Anokye Teaching Hospital, Kumasi, Ghana; 3 Division of Emergency Medicine, Faculty of Health Sciences, University of Cape Town, South Africa; 4 Emergency Medicine Directorate, Komfo Anokye Teaching Hospital, Kumasi, Ghana; 5 Department of Population, Family and Reproductive Health, School of Public Health, Kwame Nkrumah University of Science and Technology, Kumasi, Ghana; 6 Department of Emergency Medicine, University of Michigan Health System, Ann Arbor, MI, USA; 7 Department of Surgery, University of Washington, Seattle, WA, USA; 8 Department of Interdisciplinary Health Sciences, Stellenbosch University, Cape Town, South Africa

**Keywords:** Triage, Ghana, South African triage scale, diagnostic accuracy

## Abstract

**Introduction:**

The incidence of emergency conditions is increasing worldwide, particularly in low- and middle-income countries (LMICs). However, triage and emergency care training has not been prioritized in LMICs. We aimed to assess the reliability and validity of the South African Triage Scale (SATS) when used by providers not specifically trained in SATS, as well as to compare triage capabilities between senior medical students and senior house officers to examine the effectiveness of our curriculum for house officer training with regards to triage.

**Methods:**

Sixty each of senior medical students and senior house officers who had not undergone specific triage or SATS training were asked to triage 25 previously validated emergency vignettes using the SATS. Estimates of reliability and validity were calculated. Additionally, over- and under-triage, as well as triage performance between the medical students and house officers was assessed against a reference standard.

**Results:**

Fifty-nine senior medical students (98% response rate) and 43 senior house officers (72% response rate) completed the survey (84% response rate overall). A total of 2,550 triage assignments were included in the analysis (59 medical student and 43 house officer triage assignments for 25 vignettes each; 1,475 and 1,075 triage assignments, respectively). Inter-rater reliability was moderate (quadratically weighted κ 0.59 and 0.60 for medical students and house officers, respectively). Triage using SATS performed by these groups had low sensitivity (medical students: 54%, 95% CI 49–59; house officers: 55%, 95% CI 48–60) and moderate specificity (medical students: 84%, 95% CI 82 - 89; house officers: 84%, 95% CI 82 - 97). Both groups under-triaged most ‘emergency’ level vignette patients (i.e. SATS Red; 80 and 82% for medical students and house officers, respectively). There was no difference between the groups for any metric.

**Conclusion:**

Although the SATS has proven utility in a number of different settings in LMICs, its success relies on its use by trained providers. Given the large and growing burden of emergency conditions, training current and future emergency care providers in triage is imperative.

## Introduction

The incidence of emergency conditions is increasing worldwide due to aging populations, urbanization and a lack of preventative care capacity [1–3]. This increasing burden falls disproportionately on low and middle-income countries (LMICs), which are ill equipped to provide emergency care [4, 5]. Opportunely, sound planning for and organization of emergency care can prevent deaths and reduce disability without the addition of significant costs [6–8]. An important part of emergency care organization is triage [9]. Triage improves emergency care service delivery by optimizing the utilization of existing and often scarce resources among patients depending on their acuity [10]. This process is particularly important in LMICs, which often have limited human and physical emergency resource capacity [11]. However, in many LMICs, triage is under-utilized, under-resourced, and poorly researched [12]. The South African Triage Scale (SATS) was developed in 2004 as a simple triage tool for South Africa [9]. The SATS has proven reliability and validity among providers who have undergone dedicated triage training, in emergency centers with limited resources both in and out of South Africa [12]. It has become widely used in LMICs, including first-level hospitals, which are typically the first point of care for patients with emergency conditions [12, 13]. Given these successes, the Ministry of Health (MoH) of Ghana recommended that the SATS become the triage method of choice for all hospitals in the country in 2011 [14, 15]. Although it is endorsed by MoH in Ghana and is expected to be in practice, triage training for current medical school trainees and existing non-emergency medicine providers has not been delivered. Although the SATS has proven construct validity and is effective in most LMIC settings, it has not been formally assessed among providers without dedicated SATS training. In Ghana, as in many first-level hospitals in LMICs, the healthcare provider caring for patients with emergency conditions is a general practitioner (GP) or a non-physician [4, 16]. Medical students graduate, spend two years as a house-officer to become a GP, and then are deployed to first-level hospitals countrywide for at least one year. These providers are often responsible for planning and organizing triage and emergency care at their facility [17, 18]. However, emergency care training of medical students and house officers has not been prioritized [19]; thus, many house officers who are becoming GPs and preparing for their national service in first-level hospitals may lack the knowledge necessary for effective triage. To address these gaps, we aimed to: i. assess the reliability and validity of the SATS when used by providers not specifically trained in SATS use; and ii. compare triage capabilities between senior medical students and senior house officers to examine the effectiveness of our house officer training curriculum with regards to triage. The findings might inform decisions around formal triage training for current and future medical trainees in Ghana, as well as LMICs more broadly.

## Methods

### Setting

Ghana is a heavily indebted, lower-middle income country in West Africa with a population of 26 million people and an annual per capita income of US$ 1,760 [20]. This study focused on medical students and house officers at Komfo Anokye Teaching Hospital (KATH), which is a 1,200-bed tertiary facility that serves around 8.6 million Ghanaians [21]. After graduating medical school, students become house officers. House officers spend six months each in internal medicine, general surgery, obstetrics and gynecology, and pediatrics. Additionally, house officers care for patients in the Accident and Emergency Unit (A&E) at KATH who have an emergency condition that falls under the auspices of each of the specialties. Some of the concepts surrounding triage are taught as part of a broader medical and surgical curriculum for both students and house officers (e.g. shock, injury management); however, neither curriculum includes modules specifically on triage theory or methods or SATS.

### South African Triage Scale and its use in Ghana

The SATS is a physiologically based composite scoring system that includes the Triage Early Warning Score and a list of discriminators to triage patients into one of five color-coded acuity groups: Red – emergency (i.e. patient to be seen by a provider immediately); Orange - very urgent (i.e. patient to be seen by a provider within 10 minutes); Yellow - urgent (i.e. patient to be seen by a provider within 60 minutes); Green - routine (i.e. patient to be seen by a provider within 240 minutes); or Blue - dead. It was designed to be simple and used by nurses to alleviate the pressures of both human and physical resource limitations at hospitals in LMICs [12]. Given its simplicity and effectiveness in similar contexts, the MoH in Ghana designated SATS as the triage method of choice for casualty units across the country in 2011 [15]. It was incorporated into A&E care at KATH in 2010 [14]. At KATH, the nurses perform triage and patients are transported to and cared for in the respective color-coded ward. However, at the 187 first-level/district hospitals across the country, both nurses and GPs perform triage; the latter are responsible for organizing emergency care and deciding the order in which patients are resuscitated, treated, and/or referred to a higher level of care.

### Survey

We created an online survey with 25 questions via SurveyMonkey (SurveyMonkey, CA, USA) using triage vignettes validated in South Africa and Pakistan [12, 22]. Each question described a patient presentation in the form of a vignette. The type and spectrum of emergencies presented in the vignettes were similar to situations encountered in Ghana. The vignettes included information on gender, age, presenting complaint, mode of arrival to the casualty unit, and vital signs. Some vignettes also included information from investigations that are often performed at the time of triage (e.g. blood glucose, hemoglobin). Respondents were asked to assign an acuity level to each vignette that corresponded to the SATS acuity groups (e.g. emergency, very urgent, urgent, or routine); vignettes that described a patient that should have been triaged blue were not included. Each question required a response (i.e. the participant could not proceed without answering the question). To avoid testing transgressions, questions were timed and question order was randomized for each respondent. The survey was performed in English, which is the language used to teach in Ghana.

### Strategy and sample

To evaluate the survey acceptability, estimate sample size and set question times, 10 medical students and 10 house officers piloted the survey. The median medical student score was 40% and the median house officer score was 70%. Sample size was estimated to be 80 participants (i.e. 40 medical students and 40 senior house officers) using these pilot scores and:

Sample = (Z_α/2_ + Z_β_)^2^ × [p_1_(1 - μ_1_) + p_2_(1 - μ_2_)] / (p_1_-p_2_)^2^

Where Z_α/2_ is the critical value of the normal distribution at α/2 (i.e. 1.96 for a confidence level of 95%), Z_ß_ is the critical value of the normal distribution at ß (i.e. 0.84 for a power of 80%), and µ_1_ and µ_2_ are the expected proportions of correct triage responses in medical students and house officers (i.e. 40 and 70%, respectively).

A list of current senior medical students and senior house officers was obtained. Sixty randomly selected potential participants were approached from each group via both email and WhatsApp (WhatsApp, CA, USA). A link to an introduction and informed consent page was included in the messages. After providing informed consent, the survey continued to an instructions page and then the emergency vignettes.

### Reliability and validity

To assess the extent to which the triage scale yields the same assessment between different respondents rating the same patient, we measured inter-rater reliability. In accordance with the Guidelines for Reporting Reliability and Agreement Studies (GRRAS), inter-rater reliability was assessed using the unweighted, linearly weighted and quadratically weighted κ (QWK) statistic [23]. The QWK considers the degree of disagreement between responses. A weighted κ places maximum weight at the two opposite ends of the triage scale (e.g. emergency and routine); therefore, it is identical to the intra-class correlation coefficient (ICC) [10]. Thus, the ICC was not reported given its equivalence with the QWK statistic [24]. Standard errors and confidence intervals were calculated using jackknife simulation. To allow apposite comparison with other SATS assessments, point estimates for each measure of inter-rater reliability were graded using the Landis and Koch classification system as follows: 0.0 - 0.20 - slight agreement; 0.21 - 0.40 – fair agreement; 0.41 - 0.60 – moderate agreement; 0.61 - 0.80 - substantial agreement; and 0.81 - 1.00 - almost perfect agreement [25]. Ten randomly selected respondents in each of the two groups re-triaged 10 randomly selected vignettes after one week. The results of this re-assessment were used to estimate intra-rater reliability by calculating the percentage of exact agreement, as well as the percentage of agreement allowing for one level of discrepancy in the triage assignments. The triage assignment accuracy of both groups was assessed by calculating the sensitivity, specificity, and over- or under-triage relative to the triage assignment for each of the vignettes suggested by an expert triage panel. The characteristics of and methods used by the expert panel for determining the triage assignment for each of the vignettes have been previously published [22]. Briefly, a panel of 18 emergency medicine physicians and emergency nurses from both developing and developed countries independently reviewed the vignettes. Using the Delphi technique, the panel reached consensus on the ‘true’ acuity of the patient in each vignette. They assigned an acuity based on their expert opinion rather than through the direct application of SATS; however, the acuities assigned mirrored SATS colors (i.e. emergency, very urgent, urgent, and routine). By using this method, triage assignment for each vignette follows emergency care acumen, and not exclusively the application of SATS itself. Over- and under-triage were interpreted using an accepted range for average under-triage of not more than 5 - 10% and an associated average over-triage rate of 30 - 50%. These ranges are considered acceptable by the American College of Surgeons Committee on Trauma.[26] Data were analyzed using STATA v13.1 (StataCorp, TX, USA).

### Ethics

Ethical approval was obtained from the Kwame Nkrumah University of Science and Technology Committee on Human Research and Publication Ethics.

## Results

Sixty senior medical students and sixty senior house officers were approached to participate in the survey. Fifty-nine medical students (98% response rate) and 43 house officers (72% response rate) completed the survey (84% response rate overall).

### Reliability

A total of 2,550 triage assignments were included in the analysis, which consisted of 59 senior medical student and 43 senior house officer triage assignments for 25 vignettes each (1,475 and 1,075 triage assignments, respectively). Inter-rater reliability was fair in both groups, as measured by unweighted κ (Table 1). When measured by linearly and quadratically weighted κ, agreement was moderate. The level of exact intra-rater agreement among respondents was substantial (74%, 95% CI 67 – 80). When allowing for a one level of discrepancy between triage assignments, the level of intra-rater agreement increased to almost perfect (97%, 95% CI 95 - 99).

**Table 1:** Measures of inter- and intra-rater reliability of senior medical student and senior house officer triage assignments of emergency vignettes using the South African triage scale in Ghana

	Senior medical students			Senior house officers		
	Estimate	(95% CI)	Agreement*	Estimate	(95% CI)	Agreement*
κ statistic						
Unweighted	0.28	(0.20 – 0.38)	Fair	0.29	(0.21 – 0.39)	Fair
Linearly weighted	0.45	(0.34 – 0.58)	Moderate	0.45	(0.34 – 0.58)	Moderate
Quadratically weighted	0.60	(0.48 -0.75)	Moderate	0.59	(0.48 – 0.74)	Moderate
Intra-rater reliability						
Exact agreement; %	77	(69 – 85)	Substantial	74	(65 – 83)	Substantial
Agreement with one SATS level discrepancy; %	98	(95 – 100)	Almost perfect	98	(95 – 100)	Almost perfect

* Agreement according to Landis and Koch criteria

CI – confidence interval; SATS – South African Triage Scale

### Validity

Table 2 and Table 3 present the accuracy of senior medical student and senior house officer triage assignment compared with those assigned by the expert panel, respectively. Overall, the triage using SATS performed by these groups had low sensitivity (medical students: 54%, 95% CI 49 – 59; house officers: 55%, 95% CI 48 – 60) and moderate specificity (medical students: 84%, 95% CI 82 - 89; house officers: 84%, 95% CI 82 - 97). Sensitivity was lower for “emergency” and “routine” triage assignments in both groups; however, these assignments had the highest specificity. Both groups over-triaged vignette patients whose triage assignment was “routine” (medical students: 77%, 95% CI 75 – 79; house officers: 79%, 95% CI 76 – 82), and nearly half of vignette patients who were “urgent” (Table 2 and Table 3). As reference, over-triage rates should be less than 50% [26]. Alarmingly, both groups under-triaged most of the “emergency” vignette patients (80 and 82% for medical students and house officers, respectively). “Very urgent” vignette patients were under-triaged in around half of triage assignments (53 and 52% for medical students and house officers, respectively). The published and acceptable under-triage rates should be less than 10% [26].

**Table 2:** Senior medical student triage assignments of emergency vignettes using the South African triage scale compared to those of an expert panel [22]

Expert triage assignments			Senior medical student triage assignments; % (N=1,475)											
	Vignettes; n	Triage assignments; n					SATS performance versus expert panel; % (95% CI)							
			Emergency	Very urgent	Urgent	Routine	Sensitivity		Specificity		Over-triage		Under-triage	
Emergency	7	413	62*	42	12	2	44	(39 - 49)	89	(87 - 91)	-	-	80	(78 -82)
Very urgent	6	354	33	37*	22	3	42	(37 - 47)	76	(74 - 79)	20	(18 - 22)	53	(50 - 55)
Urgent	6	354	5	19	47*	18	57	(52 - 62)	79	(77 - 82)	47	(45 - 50)	23	(21 - 25)
Routine	6	354	0	2	19	77*	74	(69 - 78)	93	(90 - 93)	77	(75 - 79)	-	-
Mean							54	(49 - 59)	84	(82 - 89)	48	(46 - 50)	54	(51 - 56)

SATS – South African Triage Scale; CI – confidence interval;

* Medical student triage assignment matches the expert panel’s triage assignment

**Table 3:** Senior house officer triage assignments of emergency vignettes using the South African triage scale compared to those of an expert panel.

Expert triage assignments			Senior house officer triage assignments; % (N=1,075)				SATS performance versus expert panel; % (95% CI)							
	Vignettes; n	Triage assignments; n												
			Emergency	Very urgent	Urgent	Routine	Sensitivity		Specificity		Over-triage		Under-triage	
Emergency	7	301	65*	41	13	0	42	(36 - 47)	91	(89 - 93)	-	-	82	(80 - 84)
Very urgent	6	258	29	36*	21	7	45	(38 - 51)	75	(72 - 78)	18	(16 - 20)	52	(49 - 55)
Urgent	6	258	5	21	47*	11	61	(55 - 67)	78	(75 - 81)	48	(44 - 51)	21	(18 - 23)
Routine	6	258	0	2	19	82*	72	(66 - 77)	91	(89 - 93)	79	(76 - 82)	-	-
Mean							55	(48 - 60)	84	(82 - 87)	49	(45 - 51)	53	(51 - 56)

SATS – South African Triage Scale; CI – confidence interval

* House officer triage assignment matches the expert panel’s triage assignment

### Difference between medical student and house officer triage

There was no evidence to suggest a difference between senior medical students and senior house officers with regards to inter-rater reliability (Table 1), triage assignment accuracy, or over- or under-triage (Table 2 andTable 3).

## Discussion

This study aimed to demonstrate the reliability and validity of the SATS among providers not specifically trained in triage or SATS use and to compare medical student and house officer triage capabilities. Among untrained users, SATS demonstrated fair to moderate inter-rater reliability, low sensitivity, and moderate to high specificity. Most importantly, both groups over- and under-triaged vignette patients too frequently compared to accepted standards. Lastly, there was no difference between the capabilities of medical students and house officers to triage vignette patients correctly. These findings highlight two issues: i. while the SATS is effective in many settings, it requires some degree of specific training to be used effectively; and ii. the house officer training curriculum at our teaching facility with regards to triage is inadequate. The SATS has been used successfully in each hospital level (i.e. first-level, referral hospitals, tertiary hospitals) and numerous LMICs [12–14]. In reports from these settings, training was uniformly included as part of the SATS implementation process. For example, the SATS was successfully implemented at the Médecins Sans Frontières (MSF) hospital in Timergara, Pakistan in 2011 [12]. Like government hospitals in LMICs, this hospital also faced human and physical resource limitations. Nurses performed triage at this hospital. Prior to SATS implementation, each nurse participated in a 1-hour training about triage and SATS use specifically. After nurses had been using the SATS for at least a month, the scale was assessed in detail with methods similar to those presented here. Inter-rater reliability in that setting was moderate to substantial and intra-rater agreement was high (exact agreement 87%, 95% CI 67 – 100). Further, the sensitivity and specificity compared to the same expert panel triage assignments as we used here were significantly higher than that achieved by the medical students and house officers. In general, the nurses in Pakistan over- and under-triaged patients less often than our respondents. However, the nurses, medical students, and house officers most often under-triaged ‘emergency’ vignette patients. The differences discussed above demonstrate the importance of ensuring that staff are trained in triage theory and methods, as well as the tool they are expected to use. Despite having completed two years as a house officer and caring for patients with emergency conditions during the same period, house officers were not able to triage vignette patients more effectively than medical students. At our facility, there is no structured curriculum for triage or emergency care teaching for a house officer. House officers learn on-the-job, such as on rounds, in the clinic or operating theater, or in departmental meetings. Given that there was no difference in the triage capabilities between medical students and house officers, this approach appears to be inadequate for teaching triage fundamentals. Other LMICs have built house officer training programs that include structured modules that cover a number of topics essential to providing care in LMIC as a GP in a first-level hospital after graduation, such as triage [27]. Further, some house officer programs evaluate their trainees based upon their knowledge of the topics covered in the modules [28]; emergency care vignettes such as these might be used to assess training quality and progress over time [22, 29]. Given that other studies have reported similar triage inabilities of providers graduating to first-level hospitals in other LMICs, this is not a problem that is unique to our teaching facility [30, 31]. Thus, teaching facilities in LMICs globally should consider assessing their curricula regarding triage, particularly for providers who will soon be transferred to first-level hospitals, where they will be without senior provider supervision.

A systematic review of emergency care in 59 LMICs reported that only 17 of the countries had an emergency physician; 27 countries had non-emergency consultants providing emergency care; and 15 countries had non-physicians or medical students providing emergency care [32]. In Ghana, we have a unique opportunity to improve emergency care training by including trainees in the operations of two newly developed emergency medicine residency programs [19]. Structured emergency medicine training at these facilities is currently available to residents. At Komfo Anokye Teaching Hospital there is a two-week rotation for medical students as they rotate through surgery. However, with local and national support, this two-week “exposure” could be expanded in length and in availability. It could also be easily adjusted to include house officers. Additionally, these facilities might also serve as resources for existing providers from surrounding hospitals who might be insufficiently trained in triage or emergency care (e.g. supporting continuing education experiences). Further, advanced trainees or emergency medicine faculty might serve as trainers for existing providers in first-level and referral hospitals; such models have been effective in Papua New Guinea [33, 34]. While this study clearly demonstrates the importance of triage training and highlights current training deficiencies in our house officer curriculum, there are several limitations that should be considered when interpreting the results. First, the use of online vignettes lack patient cues and other important contextual information that can be used to improve triage accuracy. However, the vignettes and survey methods we used have been well validated in LMIC settings [12, 35]. Additionally, a published report compared the use of vignettes to live emergency patients for assessing inter-rater reliability and demonstrated an acceptable level of agreement between the two methods [36]. Second, the vignettes were developed in South Africa, which is more developed and has slightly different emergency condition epidemiology than Ghana [22, 37]. However, the vignettes were screened for contextual relevance and deemed appropriate by pilot groups of medical students and house officers. Next, the response rate of house officers was not high (72%). However, the sample frame of house officers was randomly selected, and they are all exposed to the same curriculum, or lack thereof. Lastly, some of the experts in the panel that developed consensus on the triage assignments for the vignettes were from high-income countries. These individuals might have over-assigned acuity (i.e. over-triaged vignette patients), which might partially explain why our groups under-triaged vignette patients. However, the triage assignments have been evaluated in South Africa and at a first-level MSF hospital in Pakistan with markedly lower under-triaged rates. Despite these limitations, these findings allow reasonable conclusions to be made about the importance of specific triage training for those who are to follow the SATS, as well as potential deficiencies in house officer curricula in Ghana and potentially LMICs more broadly.

## Conclusion

Although the SATS has proven utility in a number of different settings and LMICs, its success relies on its use by trained providers. Given the large and growing burden of emergency conditions, training current students and house officers in triage is imperative. A number of triage and emergency care training models exist in high-income countries, and several LMICs have formed accredited training programs. However, ways in which LMIC emergency medicine training programs can have broader benefits for future and current first-level hospital providers is yet to be seen. In the meantime, the basics of triage must be taught to medical students and house officers. Otherwise, the cadre of providers at first-level hospitals in LMICs will be unable to quickly prioritize the care of critically ill and injured patients they will certainly encounter; inadequate triage capabilities at this level may waste scarce resources and lead to avertable death and disability.

### What is known about this topic

- Triage is particularly important in LMICs, which often have limited human and physical emergency resource capacity;
- The SATS has proven utility in a number of different settings in LMICs.

### What this study adds

- The success of SATS relies on its use by trained providers;
- Given the growing burden of emergency conditions in LMICs, training current and future emergency care providers in triage is imperative.
